# Fairness in optimizing bus-crew scheduling process

**DOI:** 10.1371/journal.pone.0187623

**Published:** 2017-11-30

**Authors:** Jihui Ma, Cuiying Song, Avishai (Avi) Ceder, Tao Liu, Wei Guan

**Affiliations:** 1 School of Traffic and Transportation, Beijing Jiaotong University, Beijing, P. R., China; 2 Department of Civil and Environmental Engineering, the University of Auckland, Auckland, New Zealand; Beihang University, CHINA

## Abstract

This work proposes a model considering fairness in the problem of crew scheduling for bus drivers (CSP-BD) using a hybrid ant-colony optimization (HACO) algorithm to solve it. The main contributions of this work are the following: (a) a valid approach for cases with a special cost structure and constraints considering the fairness of working time and idle time; (b) an improved algorithm incorporating Gamma heuristic function and selecting rules. The relationships of each cost are examined with ten bus lines collected from the Beijing Public Transport Holdings (Group) Co., Ltd., one of the largest bus transit companies in the world. It shows that unfair cost is indirectly related to common cost, fixed cost and extra cost and also the unfair cost approaches to common and fixed cost when its coefficient is twice of common cost coefficient. Furthermore, the longest time for the tested bus line with 1108 pieces, 74 blocks is less than 30 minutes. The results indicate that the HACO-based algorithm can be a feasible and efficient optimization technique for CSP-BD, especially with large scale problems.

## Introduction

### Overview of CSP-BD model

Research involving public transit problems [1–6] has been done for years. It has been known for more than 50 years that the crew-scheduling problem (CSP) for bus drivers (CSP-BD) presents a bus transit company with one of its most important operational-planning problems, since crew costs usually dominate all other factors [7]. The problem involves assigning vehicle trips to crews in such a way that each trip is covered by a shift, while guaranteeing that all other duty functions are feasible and that the total cost of all duties is minimal [8–13]. Thus, under reasonable constraints, less duties means reducing more cost. Some models [7] consider the goal of minimizing the total duties under the limitation of total working hours such as 8 hours according to the legislation. While, some [14, 15] refer to the total working time to spread over for each duty in the limitation of maximum and minimum value. Some models [16–18] consider the duty cost as a whole including real cost, meal cost and so on, while sometimes [19, 20] penalty cost function is added to measure duty working hours to limit hours in labor agreement rules. Moreover, unfairness function considering different duty working hours and overtime penalty is referred in some papers [21]. However, the different idle time may also cause unfairness because more idle time means more rest time, so in this paper we add the unfairness cost function both considering the total working time and idle time for each duty, as well as the common cost and extra working hour cost functions.

### Overview of CSP-BD algorithm

CSP-BD has attracted the interest of many researchers since the 1960s, and research in this area has become more active since the 1990s. Most of the methodologies in this context are based on mathematical programming techniques or on a hybrid approach using heuristics and Integer Linear Programming (ILP) [22–25]; the success and limitations of these methodologies have been discussed in Kwan et al. (2000), and Li and Kwan (2003). In the mathematical programming-based approach, CSP-BD is formulated as a sub-set of shifts that covers all pieces of the trip, with the objective of minimizing total costs or the total number of shifts [26]. In recent years, meta-heuristics have been widely used for searching practical near-optimal solutions to NP-hard (highly complex) problems. Meta-heuristics offer three main advantages: (a) they are usually very efficient in searching through very large solution space; (b) they can result in a feasible solution; (c) each class of meta-heuristics has its own methodical and strategic structure; for example, genetic algorithms (GA), one of the most important meta-heuristics, have attracted much attention recently [14, 17, 27, 28]. In addition, effort has also been made in exploring other meta-heuristics, such as Tabu searches (TS) [19, 29, 30], simulated annealing [31] and variable neighborhood search [32].

It is worth mentioning that until now, although much research has been conducted using meta-heuristics little attention has been paid to ant-colony optimization (ACO) for CSP-BD. Even in the one notable exception, Forsyth and Wren [33], the ACO algorithm for CSP-BD is unproved, for they gave up attempting to build shifts by choosing a node in each ant move and, instead, constructed multiple shifts, relying on TRACSII [34]. The work that follows describes how to filter shifts according to certain rules. Several studies [35–37] used the ACO algorithm for air and train-crew scheduling. In addition, an ACO-based method that simulates a real ant colony with positive feedback characteristics was employed in the field of optimization to solve NP-hard problems, such as the traveling-salesman problem [38, 39].

The present paper proposes a Hybrid ACO algorithm to solve CSP-BD. First, a fully connected graph is created, attempting to search for the shortest path from the graph. A vertex represents a relief opportunity, and edges connote pieces of work. Ants move on the graph according to probabilities determined by the heuristic function and pheromone intensity. This study presents the Gamma heuristic function and one first-node choosing rule and also considers the fairness of total working time and idle time in our proposed model. The results of case studies in Beijing show that the proposed HACO performs well and can generate good quality solutions.

The following sections are organized such that the objective function with fairness of working time and idle time is described in Section 2, as well as the construction model for CSP-BD. Thereafter, the detailed design of the HACO is presented in Section 3 for solving the problem. In Section 4, two experiments are respectively to determine the best parameter combinations for HACO algorithm and to testify the sensitivity of unfair cost coefficient in the objective function. In Section 5 ten bus lines are chosen to examine the relationship of the presented costs in the objective function. Finally, conclusions are drawn in Section 6, followed by recommendations for future research.

### Crew-scheduling problem for bus drivers (CSP-BD)

The crew-scheduling problem for bus drivers (CSP-BD) involves finding a set of legal shifts or duties that can cover all trips or vehicle blocks in a particular scheduling horizon. The definition is presented in the first part of this section.

### Definitions

*Each trip* is a scheduled activity with specific starting and ending times and locations. The feasibility of a solution mainly depends on the whole available connection of several successive trips in acceptable time.

**Fig 1:**
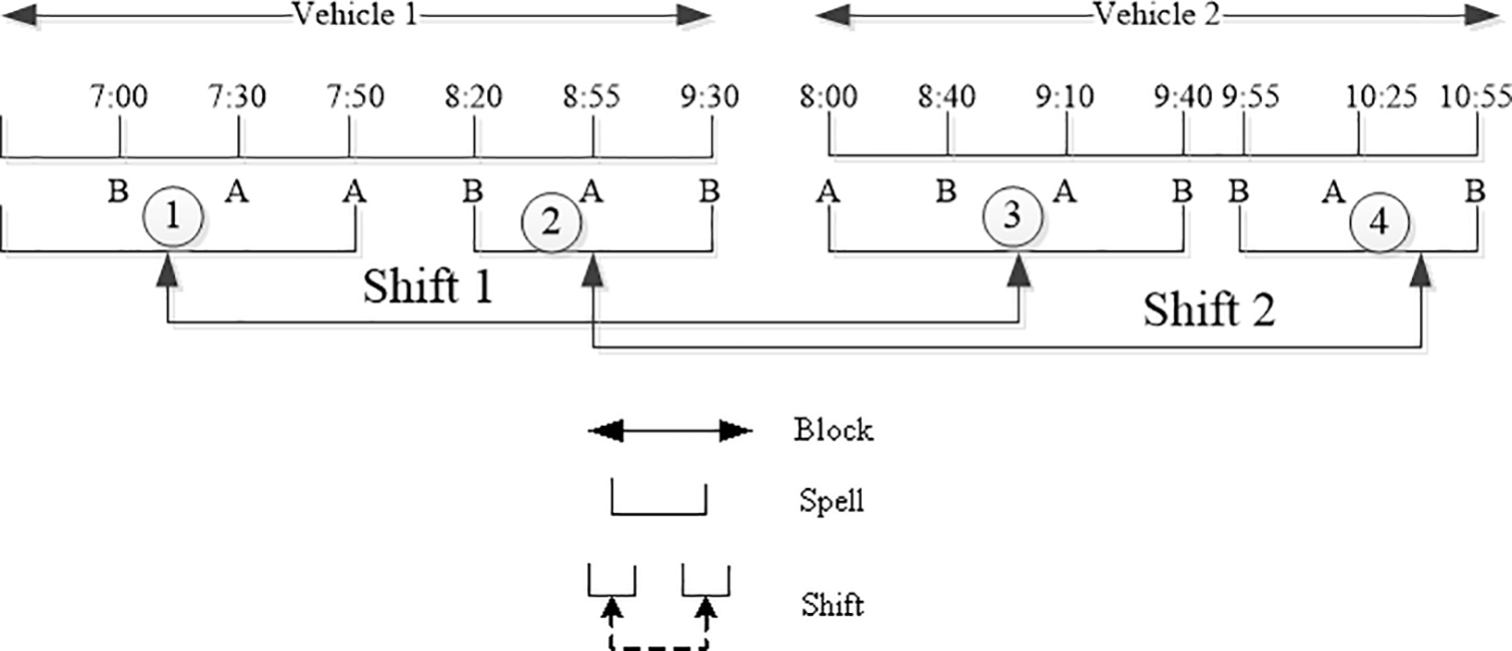
A simple illustration of blocks, spells and shifts.

*A vehicle block* illustrated in the Fig 1 may be considered a unit of work, which starts and ends at a relief opportunity (RO), meaning the time and place at which a change of drivers is possible.

*A piece of work* denotes a shorter work between two consecutive ROs completed by the same vehicle.

*The idle time* is the waiting time or rest time between the arrival time of preceding bus and the departure time of the following one at each RO.

*A driver’s shift (or duty)* is constructed by several successive pieces of work (called a spell) that can be assigned from the driver’s signing on until his/her signing off at the same depot. For example, shift 1 combines spell 1 with spell 3.

Normally, some constraints restrict crew shifts, such as labor-agreement rules that limit work hours or the time for a break, and so on. The concept of block, shift and spell is shown in Fig 1.

### Assumptions for CSP-BD

Listed below are the assumptions regarding the crew scheduling problem for bus drivers:

1. The vehicle blocks in the schedule have been utilized as input in the CSP-BD solving process;
2. The influence of different bus types can be ignored;
3. The actual total working time is allowed to exceed the total expected working time (for example, 450 minutes) that is limited in a reasonable range;
4. The duration of each RO is fixed and does not lend itself to random adjustment;
5. The time of each spell time is less than the lower bound of total expected working time (for example, 390 minutes) for an available shift.

### Objective function of CSP-BD

The objective of the CSP-BD is to minimize a bus company’s total costs, which include standard and additional salary payments to drivers, the cost of potentially unfair working time, and the cost of the average total working time and idle time. The solution is to generate *N*' legal shifts from the initial predetermined *N* shifts and *M* spells.

Following are the CSP-BD restrictions: (i) the maximum daily working time excluding overtime is 450 minutes per shift; (ii) there is an upper limit on overtime, which cannot exceed the maximum daily working time by more than 30 minutes; (iii) every crew is entitled to a maximum of 30 minutes idle time per trip; (iv) the total working time is the time span between sign-on and sign-off, consisting of driving time and idle time; (vi) idle time by definition can only begin the second after sign-on and no later than the last second of the end of the trip; (vii) for each driver there are fixed cost such as welfare and insurance, distinct from standard costs and overtime expenses. The modified equations for the crew scheduling problem, based on existing equations seen from [21], are as follows:

$$Min\;\sum_{j=1}^{N}F_{1}(v_{j})+\sum_{j=1}^{N}F_{2}(v_{j})+\sum_{j=1}^{N}F_{3}(v_{j})+N^{'}\;*\;C$$ (1)

$$s.t.\sum_{i=1}^{M}a_{ij}x_{j}\geq \lambda \;\forall j=1,2,....,N$$ (2)

$$N^{'}=\sum_{j=1}^{N}x_{j}\;\forall j=1,2,....,N$$ (3)

$$\sum_{j=1}^{N}a_{ij}x_{j}=1\;\forall i=1,2,\ldots ,M$$ (4)

$$t_{j}^{idle}=\sum_{i=1,i^{'}=1,i\neq i^{'}}^{M}t_{ii^{'}}a_{ij}a_{i^{'}j}x_{j}\;\forall j=1,2,....,N$$ (5)

$$v_{j}=\sum_{i=1,i^{'}=1,i\neq i^{'}}^{M}t_{i}a_{ij}x_{j}+t_{j}^{idle}\;\forall j=1,2,....,N$$ (6)

$$F_{1}(v_{j})=\{\begin{array}{cc} \beta_{1}*(v_{j}/60), & v_{j}\leq v^{\max}\\ \beta_{1}*(v^{\max}/60), & else \end{array}$$ (7)

$$F_{2}(v_{j})=\{\begin{array}{cc} \beta_{2}\;*\;[(v_{j}-v^{\max})/60], & v^{\max}<v_{j}\leq v^{\max}+\gamma \\ 0, & else \end{array}$$ (8)

$$F_{3}(v_{j})=\beta_{3}\;*\;[(|v_{j}-\frac{\sum_{j=1}^{N}v_{j}}{N}|+|t_{j}^{idle}-\frac{\sum_{j=1}^{N}t_{j}^{idle}}{N}|)/60]$$ (9)

$$a_{ij}\in (0,1),\forall i=1,2,\ldots ,M,j=1,2,....,N$$ (10)

$$x_{j}\in (0,1)\;\forall j=1,2,....,N$$ (11)

*F*_1_(*v*_*j*_): Common cost within the maximum constraint time (Yuan);
*F*_2_(*v*_*j*_): Extra cost that is the penalty cost for the extra working time (Yuan);
*F*_3_(*v*_*j*_): Unfair cost that is the penalty cost for deviating from the average total working time and total idle time in a shift (Yuan);
*N*': The total number of generated shifts in a solution;
*C*: The fixed cost coefficient, such as welfare and/or insurance for each driver that is set as 70 Yuan;
*N*'*C: Fixed cost that is the sum cost of all generated shifts;
*a*_*ij*_: The variable that is equal to 1 when spell *i* is selected for shift *j*, otherwise *a*_*ij*_ is equal to 0;
*x*_*j*_: The variable which is equal to 1 when shift *j* is selected otherwise *x*_*j*_ is equal to 0;
*λ*: The minimum number of spells in a shift set as 1 in the following examples;
$t_{j}^{idle}$: The total idle time in a shift (minute);
$t_{ii^{'}}$: The connecting time between spell *i* and *i*' ranging from (0,30] minutes that equals 0 when it is out of that range(minute);
*v*_*j*_: The total working time in a shift (minute) including the sum of spell time and idle time;
*t*_*i*_: The total time of a spell *i* (minute);
*v*^max^: Maximum constraint working time for the standard fixed cost (Yuan) set as 450 (minute) in the following examples;
*β*_1_: Common cost coefficient equals normal salary per hour (Yuan per hour) set as 15;
*γ*: The maximum extra time beyond *v*^max^ (minute) set as 30 (Yuan);
*β*_2_: Extra cost coefficient (Yuan per hour) set as 30 (Yuan) twice of the common cost coefficient;
*β*_3_: Unfair cost coefficient means the penalty cost for deviation from the average total time and idle time per hour (Yuan per hour);

The object of the Eq (1) is as a function to minimize the total cost, including the common cost within the maximum constraint time, the extra cost for overtime, the fairness cost for deviating from the average total working time, and the total idle time in a shift and extra fixed costs incurred by an increase of one shift. Eqs (2) and (3) respectively stand for the amount of spells in a shift and the amount of shifts in a solution, and Eq (4) indicates that each spell is only selected by one shift. Eqs (5) and (6) are respectively the calculation of total idle time and working time in a shift. Eq (7) is the common cost multiplied by total working time within its constraints. Eq (8) is the extra cost paid for overtime work when the total working time exceeds the maximum working time according to regulation. Generally, the extra cost paid per hour is much higher than the common cost per hour. In this case, the extra cost paid per hour is twice of the standard salary per hour. Eq (9) is the unfair cost for deviation from the average level working time and idle time. It reflects consideration for fairness in relation to drivers’ working time and availability of the schedule.

## HACO-based algorithm for CSP-BD

The common ACO algorithm was inspired by the foraging behavior of ant colonies, which find the shortest route between the ants’ nest and a source of food by exchanging information via pheromone trails left behind by each ant on a trip. Over time, however, the pheromone trail starts to evaporate, thus reducing its attractive strength. The more time it takes for an ant to travel across the path and back again, the more time the pheromones along the path have to evaporate. A short path, by comparison, is marched over more frequently, and thus the pheromone density on shorter paths obviously becomes higher than on longer paths. Thus, when one ant finds a good (i.e., short) path from the colony to the food source, other ants are more likely to follow that path, and positive feedback eventually leads to all the ants’ following a single path.

We note some similarities between the CSP-BD and the traveling salesman problem (TSP) as a means of further substantiating the case for the algorithm proposed in this paper. In the well-informed TSP, each node represents a city to be traversed in iteration, and link values are distances between nodes. Likewise, CSP-BD uses spells as path nodes, and the nodes can be connected by links according to idle time restrictions and consideration for arrival at or departure from a depot.

Accordingly, the characteristics of CSP-BP are integral to our proposed hybrid ant-colony optimization algorithm (HACO) based on Maximum and Minimum Ant System (MMAS). This section primarily describes the process of designing the HACO-based algorithm for solving the CSP-BD problem. Naturally, this includes the node-choosing rule in formulating the probability function, as well as detailed updating of the pheromone-trails rules in MMAS; it is performed through constructing a selection function and generating shifts rules, both are critical procedures of the algorithm.

### HACO: Node-choosing rule

Prior to examining the process of ants start searching for a route, it is essential that we identify certain indispensable variables.

*T*_1×*M*_: A Tabu table deposits the nodes that have been visited in a searching route and its condition at initialization is to empty the table upon the beginning of an ant’s traversing.
*C*_*M*×*M*_: A correlation matrix reflects the relation between two nodes (spells). In TSP, the values of the correlation matrix are the distances between two cities. However, in CSP-BD, the values are recognized as the idle time between two nodes under the given constraints, such as maximum idle time, arrival or departure depot and maximum working time in a shift. For example, if the arrival depot of spell *i* is the same as the departure depot of spell *i*', then the idle time for the two spells and their total working times are less than the maximum restrictions, such that the value of $C_{ii^{'}}$ is equal to the idle time between spell *i* and spell *i*'. From the correlation matrix, each node corresponds to its potential connecting node indicated in the matrix row.
*H*_*M*×*M*_: The heuristic matrix shows data results transformed by the heuristic function. Different forms of the heuristic function are listed below.
*P*_*M*×*M*_: The pheromone matrix displays the pheromone intensity on the search route. The process of initiation is simplified based on the correlation matrix, whereas pheromone intensity remains at zero on the positions with zero value in the correlation matrix. The matrix updates after all ants traverse their routes in a cycle.

### The next node-choosing rule

In the iterative process of the ants’ choosing the next nodes, transition rules play an important role until all nodes have been selected from the graph. Transition rules with many equations and even more parameters stands on the current node to select the next node from the unselected node pool depositing the unvisited nodes in the process of constructing a new shift. Here, the next node is not only the one that may depart later than the arrival time of current node but also the one that arrive earlier than the current node. Then the searching range enlarges compared with the only searching the nodes that depart later.

$$j=\{\begin{array}{cc} \arg\;\max\{[\underset{u\in allowed_{it}^{p}}{\tau_{iu}(t){]}^{\alpha }}.\underset{u\in allowed_{i}^{c}}{{\left[\eta_{iu}\right]}^{\beta }}\} & q\leq q_{0}\\ S & else \end{array}$$ (12)

*τ*_*iu*_(*t*): Pheromone intensity of the trail between node *i* and node *u* in the process of *t*^*th*^ iteration. Its value is reflected on the pheromone matrix and it increases or decreases according to the number of ants traversing the trail;
$allowed_{it}^{p}$: The non-empty node set for unselected node *i* in the corresponding row or column of the pheromone matrix in the process of *t*^*th*^ iteration;
$allowed_{i}^{c}$: The non-empty node set for unselected node *i* in the corresponding row or column of the correlation matrix;
*η*_*iu*_: The heuristic function presenting the closeness between node *i* and node *u*; it is inversely proportional to the idle time $t_{iu}^{idle}$. Here, if *η*_*iu*_ = 0, that means node *u* may not exist in $allowed_{i}^{c}$.

From the property of the problem, the larger the idle time, the less closeness that exists between node *i* and node *u*. Gamma-Function (G-F) functions are considered to be the fittest function seen from Eq (13).

$$\begin{array}{l} \eta_{iu}=\{\begin{array}{cc} \omega {\left(t_{iu}^{idle}\right)}^{-\lambda_{1}}e^{(-\lambda_{2})t_{iu}^{idle}} & t_{iu}^{idle}>0\\ 0 & else \end{array}\;\forall u\in allowed_{i}^{c},\lambda_{1}>0,\lambda_{2}>0,\omega >0 \end{array}$$ (13)

*α* and *β* define the importance of pheromone intensity *τ*_*iu*_(*t*) versus the heuristic function. Here, both *α* and *β* are non-negative.

*q*: a random number uniformly distributed from 0 to 1, chosen from the *rand* function in MATLAB.
*q*_0_: a parameter determining the degree of correlation of exploitation compared with exploration. Exploitation means acquiring an exact number or value through iterative analysis and calculation, however, exploration is the result of repeated attempts with randomness.

If *q* ≤ *q*_0_, the unique node with the maximum value in Eq (12) is chosen according to exploitation. On the other hand, the parameter is exploration-oriented when the next node is chosen according to S, which is a random variable selected according to the probability obtained by:

$$p_{ij}^{k}(t)=\{\begin{array}{cc} \frac{{\left[\tau_{ij}(t)\right]}^{\alpha }.{\left[\eta_{ij}\right]}^{\beta }}{\sum_{\begin{array}{l} u\in allowed_{it}^{p}\\ u\in allowed_{i}^{c} \end{array}}{\left[\tau_{iu}(t)\right]}^{\alpha }.{\left[\eta_{iu}\right]}^{\beta }} & j\in allowed_{it}^{p},j\in allowed_{i}^{c}\\ 0 & otherwise \end{array}$$ (14)

### First node-choosing rule

Because of the limitation for total working time, more than one shift is contained in a solution. When one shift is completed, the next chosen node may have no direct relationship with the current chosen node. Then, once one shift has been selected, the next problem concerns how to choose the first node in the next shift. Here, similar rules for choosing the first node for each shift as the transition rules are proposed.

$$j^{'}=\{\begin{array}{cc} \underset{\begin{array}{l} i\in unselected_{t}^{k}\\ u\in allowed_{it}^{p} \end{array}}{\arg\;\max\{\tau_{iu}(t)\}} & q^{'}\leq q_{0}^{'}\\ r & otherwise \end{array}i=1,2,\ldots ,n$$ (15)

$unselected_{t}^{k}$: The non-empty node pool that has not been visited until now at *t*^*th*^ iteration for the *k*^*th*^ ant, *q*' is the same meaning as *q*, and $q_{0}^{'}$ defines the relative importance of exploitation versus exploration, similar to *q*_0_ above mentioned. If $q^{'}\leq q_{0}^{'}$, the node chosen is said to be exploitation-oriented; that is, the unselected node with the highest pheromone density at *t*^*th*^ iteration is chosen. In contrast, the exploration defines that the next node is selected randomly from *unselected*_*k*_ using the *rand* function in MATLAB. The choosing rules do not contain the heuristic function *η*_*ij*_ for the reason that there exists no connecting nodes defining in the correlation matrix and has less of relationships compared with the prior nodes in the Tabu table.

### HACO: Updating the pheromone trail rule in MMAS

The updated trail rule in MMAS is that the best ants globally or only the best ants deposit pheromones after each cycle. The range of the quantity of pheromone is limited to [*τ*_min_,*τ*_max_] in order to avoid staggering in the iteration; *τ*_min_ and *τ*_max_ denote the minimum and maximum pheromone trail intensity, respectively. This rule is formulated as:

$$\tau_{ij}(t+1)=(1-\rho )\tau_{ij}(t)+\Delta \tau_{ij}^{best}(t,t+1)$$ (16)

$$\Delta \tau_{ij}^{best}(t,t+1)=\frac{Q}{Cost(A_{gbest})}$$ (17)

*ρ*: The quantity of evaporation (0 < *ρ* < 1). All the pheromone intensities will evaporate in various degrees in order to avoid repeating the same path;
(1−*ρ*).*τ*_*ij*_(*t*): The remnant of pheromone quantity;
$\Delta \tau_{ij}^{best}(t,t+1)$: Quantity of pheromones deposited on the link between node *i* and node *j* by the ant that constructed a path with the least cost at *t*^*th*^ iteration;
Q: Control factor for a pheromone to avoid a cost value that is too large to converge to the local optimal point early or too small to search randomly and is set as 10 in the following experiments;
Cos*t*(*A*_*gbest*_): Global minimum cost from the beginning until the present.

The updated trail rule effectively directs the ants to the most promising space, which rapidly becomes the relatively optimal path.

### HACO: Generating shifts rule

Based on the above rules, several nodes have been selected to construct an intact shift. However, due to the constraints on the total working time *v*_*j*_, the length of a shift is also limited. Here, four conditions and their solutions are proposed according to the maximum total working time *v*^max^ and minimum total working time *v*^min^.

$$v_{j}<v^{\min}$$

On the condition that the total working time for one shift is less than the minimum working time, if the following node in $allowed_{it}^{p}$ and $allowed_{i}^{c}$ for this shift exists, then a new node is added to the shift, otherwise, the uncompleted shift is considered as a new shift.

$$v^{\min}\leq v_{j}\leq v^{\max}$$

If the total working time is within such range, a new intact shift is generated. The nodes are placed in the Tabu Table, and then preparations can be made for the next shift.

$$v^{\max}<v_{j}\leq v^{\max}+\gamma$$

If the total working time of this shift satisfies the above range, then a new shift with the extra working time will be generated, calculating the overtime costs for this shift.

$$v_{j}>v^{\max}+\gamma$$

If the total working time exceeds the maximum extra working time, i.e., the working time for the new, added spells is not adapted to this shift, then delete the new added node and change another feasible spell. If no spell node is available for this uncompleted shift, then this short shift will also be generated as a new shift.

### Search procedure for solutions using the HACO algorithm

**Fig 2:**
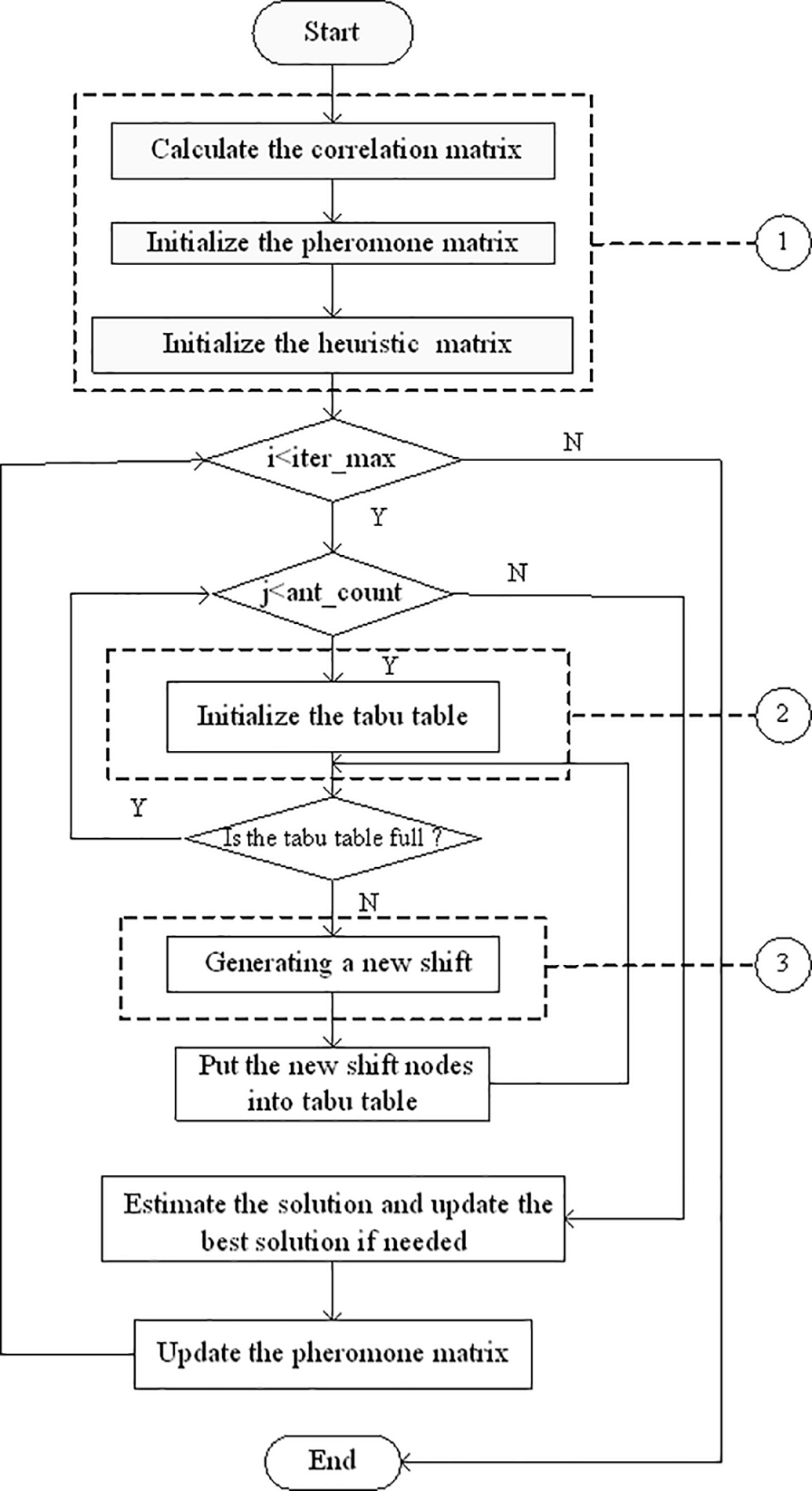
Search procedure using the HACO algorithm. The first part of procedure expressing the initialization at the beginning of algorithm. (b) the second part expressing the updating process. (c) the third part expressing the key process of the algorithm.

The main task of the HACO algorithm in solving the CSP-BD is to model the objects in the search for the shortest path along a weighted graph with constraints. Calculations are then made in the iterative process, and probabilities are generated as to where to move next, based on pheromone densities and closeness levels. Some constraints are provided to limit the alternative sets, and appropriate parameters of the probability equation are chosen by trial and error. The search procedure for the HACO-based algorithm is shown in Fig 2. The following three main processes are illustrated in detail:

### Initialization for three matrixes

Upon beginning the procedure for the HACO algorithm in solving the CSP-BD, some data structure such as correlation matrix, pheromone matrix and heuristic matrix are defined in order to store original and changing data. The correlation matrix stores the original spell data relationship and it remains unchangeable throughout the procedure and provides support for most of the following matrixes. If spell node *i* coincides the depot and idle time constraints, the matrix is filled with the idle time number; otherwise, it is filled with zero. The pheromone matrix is consisted of pheromone density and changes its values in the iterative process. The initiation of this matrix is on the basis of updating rules; all values in the matrix are at the minimum limit. The heuristic matrix is generated based on the correlation matrix, for the only variable in the heuristic function is idle time reflected in the correlation matrix.

### Initialization for Tabu table

Before each ant starts to move, the Tabu Table, which is regarded as a node pool with the visited node, is empty. Thereafter, any visited node is put into the pool until the pool is full. Ultimately, when an ant has traversed all routes, all visited nodes are placed into the pool.

## Generating a new shift

**Fig 3:**
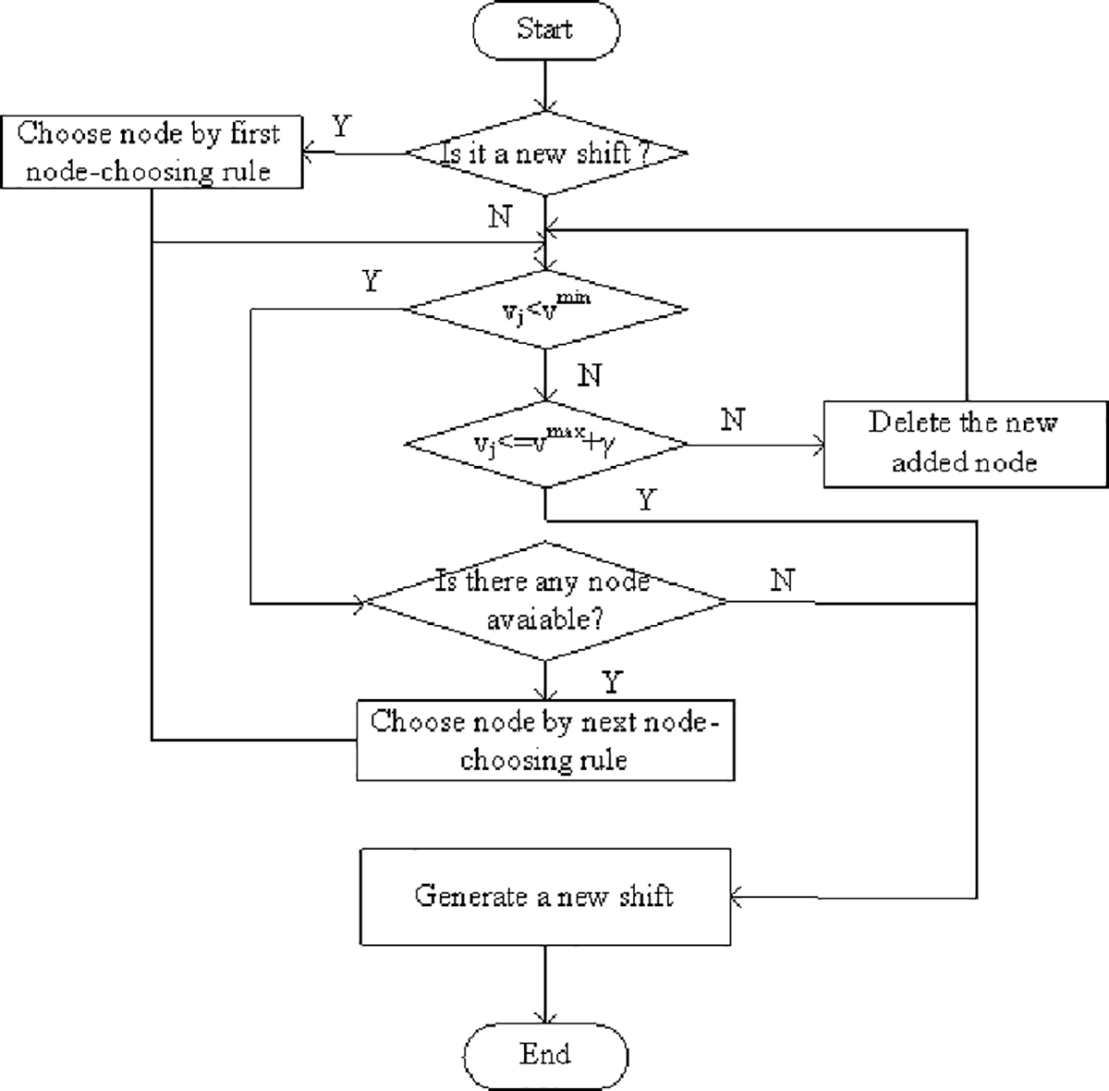
Procedure for generating a new shift in the HACO algorithm.

The procedure combines the rules specified above, to generate a new shift, as shown in Fig 3. First, generate a new undetermined shift according to various node choosing rules. Then, the total working time of the new shift coincides the minimum and maximum working time limit. We are then referred to some chosen conditions: a) the total working time confines to minimum and maximum working time; b) the total working time is more than the maximum working time yet less than the allowed maximum overtime; c) when the total working time is less than the minimum working time, but no available nodes satisfy the current spell, then the spell can also be considered as a new shift.

This figure illustrates the whole procedure of HACO algorithm in detail. At first, the structure of the main data is illustrated: the correlation matrix, the pheromone matrix and the heuristic matrix. If the iteration *i* is less than the original maximum number *iter*_max, then the ant number *j* is determined, and otherwise, the whole algorithm finishes. Then the Tabu table is initiated and a new shift is generated if the Tabu table is not full. The new shift nodes are put into the Tabu table until it is full, that is, all the nodes are put into the Tabu Table. If all ants finish their routes, all solutions are estimated by calculating the total cost and storing the best one. The pheromone density is updated in the pheromone matrix. Then application of the algorithm and the search for the best solution is continued.

## Computational tests

To test the performance of the proposed HACO for CSP-BD, two main computational experiments were made. The first experiment was to determine the best parameter combinations for this algorithm. The second experiment was to testify the sensitivity of unfair cost coefficients. The computational tests of the HACO algorithm were carried out by applying the code in MATLAB.

### Parameter settings of HACO

The object of this section is to analyze the performance of HACO with different parameter settings for CSP-BD instance with respect to bus line 26 with two depots, 44 blocks and 294 pieces of work with the heuristic Gamma Function (G.F) in which the parameters are set as *λ*_1_ = 0.5, *λ*_2_ = 0.05 and the constant coefficients in objective function are set as *β*_1_ = 15, *β*_2_ = 30, *β*_3_ = 7.5 (Yuan) and the minimal and maximal quantity of pheromone are set as *τ*_min_ = 0.005, *τ*_max_ = 3 (minute). The most important parameters considered in HACO include (a) exploration threshold *q*_0_ or $q_{0}^{'}$, (b) the ratio of *α*:*β* (the relative importance of pheromone trails versus the heuristic function), (c) trace persistence coefficient 1−*ρ*. This study briefly examines the influence of these parameters on HACO with respect to three criteria: the minimal and average minimal cost in each iteration denoted by M.C. and A.M.C. and the number of the iterations where the minimal cost is less than the total average minimal cost defined as Num. Then, we determine the best combination of parameters for achieving the best results. Performance by different combinations of parameter values is tested according to a series of experiments. The various values for each parameter are *q*_0_ = 0.8,0.9,0.95, *α*:*β* = 1:1,1:2,1:5, *ρ* = 0.1,0.05,0.01. Each parameter combination in Table 1 was run 20 times and there were 50 ants for each trial. The results are presented in Table 2. From the obtained results, obviously, this combination, q_0_ = 0.9, *α*:*β* = 1:5, 1−*ρ* = 0.9, could get the best results with minimum cost, minimum average cost and maximum number.

**Table 1:** Different parameter combinations.

NO.	*q*_0_	*α*:*β*	1−*ρ*	NO.	*q*_0_	*α*:*β*	1−*ρ*	NO.	*q*_0_	*α*:*β*	1−*ρ*
**1**	0.8	1:1	0.9	**10**	0.9	1:1	0.9	**19**	0.95	1:1	0.9
**2**	0.8	1:1	0.95	**11**	0.9	1:1	0.95	**20**	0.95	1:1	0.95
**3**	0.8	1:1	0.99	**12**	0.9	1:1	0.99	**21**	0.95	1:1	0.99
**4**	0.8	1:2	0.9	**13**	0.9	1:2	0.9	**22**	0.95	1:2	0.9
**5**	0.8	1:2	0.95	**14**	0.9	1:2	0.95	**23**	0.95	1:2	0.95
**6**	0.8	1:2	0.99	**15**	0.9	1:2	0.99	**24**	0.95	1:2	0.99
**7**	0.8	1:5	0.9	**16**	0.9	1:5	0.9	**25**	0.95	1:5	0.9
**8**	0.8	1:5	0.95	**17**	0.9	1:5	0.95	**26**	0.95	1:5	0.95
**9**	0.8	1:5	0.99	**18**	0.9	1:5	0.99	**27**	0.95	1:5	0.99

**Table 2:** Results for different parameter combinations of Line 26 (the given shift number is 72 in real life made by manual work).

NO.	M.C	A.M.C	NUM	NO.	M.C	A.M.C	NUM
**1**	12859.89	13127.01	2	15	13114.51	13319.46	0
**2**	12963.46	13186.77	1	**16**	**12749.62**	**13080.98**	**5**
**3**	13053.49	13178.98	0	17	13047.45	13311.54	0
**4**	12997.55	13117.31	0	18	13047.49	13244.93	0
**5**	12926.76	13154.14	3	19	13022.01	13203.37	0
**6**	12942.59	13178.70	2	20	12924.01	13125.69	1
**7**	12885.50	13064.93	3	21	12938.31	13176.17	1
**8**	12823.80	13170.35	2	22	12803.76	13074.32	4
**9**	12840.93	13176.08	2	23	12956.78	13126.53	1
**10**	12899.75	13145.55	2	24	13094.72	13239.91	0
**11**	13110.63	13345.84	0	25	12855.44	13072.66	4
**12**	13103.07	13339.54	0	26	13083.99	13198.50	0
**13**	13164.47	13307.56	0	27	13024.18	13219.16	0
**14**	12954.44	13186.26	1				

### Experiments with the unfair cost coefficient

**Fig 4:**
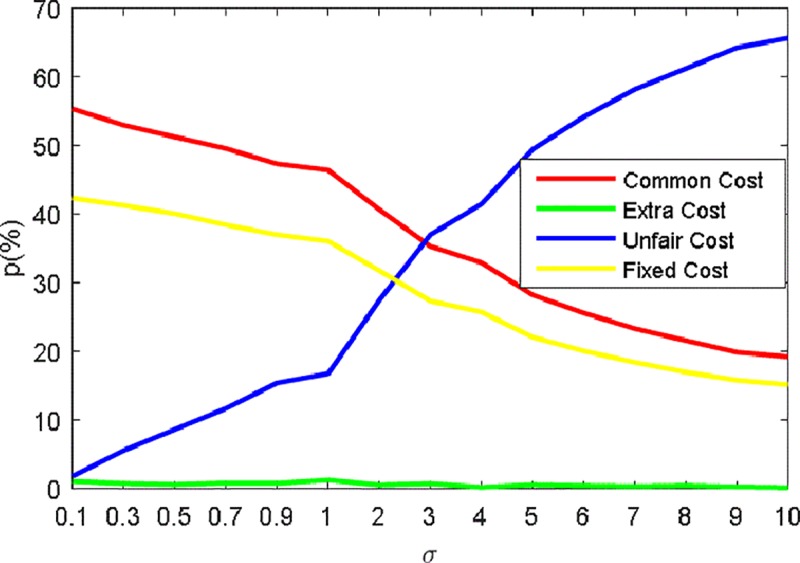
The occupancy of four basic costs at different *σ* for #26. (A) *σ* expressing the ratio between the chosen value and the common cost coefficient. (B) four basiccosts expressing respectively common cost, extra cost, unfair cost and fixed cost. (c) p expressing the occupancy ratio of each cost.

In this section, we choose a group of values for the unfair cost coefficient *β*_3_ in Eq (1) to determine the fairness in CSP-BD. The chosen value of *β*_3_ refers to the common cost coefficient *β*_1_, that is, *β*_3_ = *σβ*_1_. The range of value for *σ* we define in this part includes two sets: *R*_1_ = {0.1,0.3,0.5,0.7,0.9} and *R*_2_ = {1,2,3,4,5,6,7,8,9,10}. These experiments are based on bus line 26 with two depots, 44 blocks and 294 pieces of work with the heuristic Gamma Function (G.F) in which the parameters are set as *λ*_1_ = 0.5, *λ*_2_ = 0.05 as well as q_0_ = 0.9, *α*:*β* = 1:5, 1−*ρ* = 0.9. The maximum iteration is set as 200 and the number of ants is 50. The experiment results with the respective minimal total cost are listed in Table 3 with various costs represented in the objective function equation. From Table 4, we may safely observe the changes of involved costs with the growth of *σ*. Except for the clear increase of the minimal total cost and the unfair cost, the common cost changes little and the extra cost relatively deceases. Besides, the fixed cost seen from the number of duties also grows. The unfair cost obviously outweighs other three costs when *σ* is greater than 2. Furthermore, we also calculate the occupancy p of each cost in total cost in order to clearly compare the changes when the given parameter *σ* grows. In Fig 4, we can clearly see that both the occupancy of common cost and fixed cost obviously decrease while the occupancy of unfair cost increases significantly especially when the given parameter *σ* is greater than 1. Moreover, we also see that the extra cost changes little that shares a small proportion of the total cost.

**Table 3:** Results for HACO with minimization in each iteration with *σ* ranging from 0.1 to 10; the unit for all cost is Yuan in the following tables.

*σ*	Total Cost	Common Cost	Extra Cost	Unfair Cost	Shifts	Fixed Cost
**0.1**	11902.43	6587	128	217.43	72	5040
**0.3**	12382.35	6559.25	94	689.10	73	5110
**0.5**	12826.73	6551.25	101.5	1133.98	73	5110
**0.7**	13294.21	6589.25	109.5	1555.46	73	5110
**0.9**	13809.53	6535.75	107	2126.78	73	5110
**1**	14172.52	6579.25	178	2375.27	73	5110
**2**	16080.55	6541.75	85.5	4413.30	73	5110
**3**	18690.25	6598.25	134	6918	73	5110
**4**	19848.81	6535.5	41.5	8231.81	73	5110
**5**	23150.71	6536	129.5	11445.21	73	5110
**6**	25820.16	6610.25	110.5	13989.41	74	5180
**7**	28151.66	6565.25	94	16382.41	74	5180
**8**	30505.61	6580.75	134	18680.86	74	5180
**9**	32781.84	6515.75	86	21070.09	74	5180
**10**	34215.04	6576.5	55.5	22473.04	74	5180

**Table 4:** Ten bus lines with respective blocks and pieces.

Line	Blocks no.	Pieces no.
**#306**	18	144
**#695**	36	224
**#348**	16	241
**#26**	44	294
**#43**	25	296
**#467**	18	310
**#28**	27	378
**#34**	24	362
**#345**	72	628
**#322**	74	1108

### Experiment results

The conditions and best results of the improved ant-colony optimization algorithm are presented, based on the above experiments. The database for these computational tests was the Beijing Bus Transit Group, one of the largest bus transit companies in the world with more than 10,000 buses. We chose 10 bus lines with two depots from database that was shown in Table 4. The parameters for HACO algorithm were q_0_ = 0.9, *α*:*β* = 1:5, 1−*ρ* = 0.9, using the Gamma heuristic function and the iteration number was 200 and the number of ants was 50. Experiments with *σ* = 1, *σ* = 2, *σ* = 5 were respectively done with the ten represented lines. Other parameters were the same with the above experiments. The final solution was derived from the average minimum results in these iterations. All experiments were run with Intel Core i5-3570 CPU and 4 G installed memories (RAM).

The results are displayed in Tables 5–7 with three following tables. From those tables, it verifies once again that the changes of common cost, extra cost and fixed cost had no direct relationship with unfair cost for there is no obvious increasing or decreasing in those referred costs even though the increase of unfair cost ranges from several to several dozen times. Furthermore, we may clearly see that the common cost, unfair cost and fixed cost are relative to the respective bus lines. For example, all of those provided cost for #345 are the highest, in contrast, cost for #348 are the lowest. The value of common cost, unfair cost and fixed cost are almost the same when the given parameter *σ* is equal to 2. The running time for each line was also listed in the last column and the longest running time is #322 with the most pieces and blocks. More experiments will be done in the future to test the relationship of the coefficients of common, unfair and fixed cost. The extra cost, in our experiments is relatively low compared with other cost for those chosen timetables made by dispatcher are strictly confined in the given constraints of total working time.

**Table 5:** Results for three experiments for *σ* = 1.

*σ* = 1	Total cost	Common Cost	Extra Cost	Unfair Cost	Fixed Cost	Time(s)
**#306**	6221.72	2428.75	3.5	1339.47	2520	28.193
**#695**	13265.7	5775	0	2310.7	5250	52.474
**#348**	5326.05	2130	0	1096.05	2170	47.363
**#26**	14172.52	6579.25	178	2375.27	5110	79.621
**#43**	12228.9	4425.25	10	2893.66	4970	86.257
**#467**	7411.86	3402.25	0	1209.61	2870	85.352
**#28**	10681.35	4130.25	0	2491.10	4130	109.35
**#34**	9520.45	4035.25	0	1705.2	3850	95.07
**#345**	27814.14	10998.25	3.5	5822.39	11060	500.02
**#322**	26317.73	9736.25	0	5661.48	10990	1690.16

**Table 6:** Results for three experiments for *σ* = 2.

*σ* = 2	Total cost	Common cost	Extra cost	Unfair cost	Fixed Cost	Time(s)
**#306**	7936.20	2412.25	0	2863.95	2730	30.655
**#695**	15080.49	5739.75	0	4300.74	5110	56.508
**#348**	6547.84	2130.25	0	2247.59	2240	47.044
**#26**	16080.55	6541.75	85.5	4413.30	5110	80.821
**#43**	14792.61	4424.5	0	5468.11	4970	76.114
**#467**	8964.39	3376.75	0	2717.64	2940	69.813
**#28**	13081.97	4207.75	0	4884.22	4060	100.758
**#34**	11201.29	4030.25	0	3391.04	3850	92.647
**#345**	33270.12	11025	4	11531.12	10780	500.805
**#322**	31746.59	9806	0	11160.59	10850	1606.352

**Table 7:** Results for three experiments for *σ* = 5.

*σ* = 5	Total cost	Common cost	Extra cost	Unfair cost	Fixed Cost	Time(s)
**#306**	11908.42	2440	**0**	6878.42	2660	30.433
**#695**	20667.21	5763.25	**0**	9933.96	5040	53.533
**#348**	9582.41	2128.5	**0**	5283.91	2240	51.932
**#26**	23150.71	6536	129.5	11445.21	5110	85.584
**#43**	23421.83	4414	0.5	13967.33	5110	86.791
**#467**	13370.82	3416.5	0	6944.32	3080	67.534
**#28**	19665.11	4129.25	0	11545.86	4060	98.412
**#34**	16303.25	4056.75	0	8466.5	3850	91.975
**#345**	51328.53	10945.75	0.5	29322.28	11130	483.396
**#322**	47439.55	9773.5	0	27026.05	10710	1647.356

## Conclusion

In this work, we consider the fairness of total working and idle time in the process of solving bus-crew scheduling problem using a powerful HACO based algorithm. A series of experiments were done successively in order to determine the best combination of HACO algorithm and validate the sensitivity of the unfair coefficient. From the results, we learned that the common cost, fixed cost and extra cost had no direct relationship with the unfair cost for the values of those three costs were basically unchanged when the unfair cost increased. Except for extra cost, the values of other three costs were very close when the unfair coefficient was at twice the coefficient of common cost. Furthermore, both the occupancy of common cost and fixed cost decreased while unfair cost occupancy deceased. In the last experiments, we chose ten bus lines using real-life cases from the Beijing Public Transport Holdings (Group) Co., Ltd. with pieces and blocks from lowest to highest under three unfair coefficients to examine the relationship of four given cost in the objective function. The final results showed that the common cost associated with the fixed cost for both of the two costs related to the generated duties. The unfair cost for all of bus lines approached to common and fixed cost when the unfair coefficient was twice as much as that of common cost. For the extra cost with small value, it seemed to relate indirectly to other costs. In addition, the results also clearly verified that HACO algorithm performed noticeably advantages, especially for larger scale problems. In our experiments, the bus line with 1108 pieces, 74 blocks consumed less than 30 minutes in an acceptable time. More experiments will be done in the future to test the relationships of the coefficients of common, unfair and fixed cost. In addition, it is worth noting that changing cost parameters may exert an effect on the solution in cases in which serval costs and constraints parameters of the CSP-BD are set as constants. Therefore, more attention should be paid to cost parameters in future works. A further study that is underway will add more constraints to the problem, such as meal time, driver or vehicle constraints and so on.

## Supporting information

S1 Appendix The timetable for Line #26. (XLS)

S2 Appendix The timetable for Line #28. (XLS)

S3 Appendix The timetable for Line #34. (XLSX)

S4 Appendix The timetable for Line #43. (XLSX)

S5 Appendix The timetable for Line #306. (XLS)

S6 Appendix The timetable for Line #322. (XLS)

S7 Appendix The timetable for Line #345. (XLS)

S8 Appendix The timetable for Line #348. (XLSX)

S9 Appendix The timetable for Line #467. (XLSX)

S10 Appendix The timetable for Line #695. (XLS)
